# Analysis of dog breed diversity using a composite selection index

**DOI:** 10.1038/s41598-023-28826-3

**Published:** 2023-01-30

**Authors:** Wei-Tse Hsu, Peter Williamson, Mehar Singh Khatkar

**Affiliations:** 1 Sydney School of Veterinary Science, Faculty of Science, The University of Sydney, Sydney, NSW 2006 Australia; 2 School of Animal and Veterinary Science, Faculty of Sciences, University of Adelaide, Roseworthy, SA 5371 Australia

**Keywords:** Evolution, Genetics

## Abstract

During breed development, domestic dogs have undergone genetic bottlenecks and sustained selective pressures, as a result distinctive genomic diversity occurs to varying degrees within and between breed groups. This diversity can be identified using standard methods or combinations of these methods. This study explored the application of a combined selection index, composite selection signals (CSS), derived from multiple methods to an existing genotype dataset from three breed groups developed in distinct regions of Asia: Qinghai-Tibet plateau dogs (adapted to living at altitude), Xi dogs (with superior running ability) and Mountain hounds (used for hunting ability). The CSS analysis confirmed top ranked genomic regions on CFA10 and CFA21 in Qinghai-Tibet plateau dogs, CFA1 in Xi dogs and CFA5 in Mountain hounds. CSS analysis identified additional significant genomic regions in each group, defined by a total of 1,397, 1,475 and 1,675 significant SNPs in the Qinghai-Tibetan Plateau dogs, Xi dogs and Mountain hounds, respectively. Chitinase 3 Like 1 *(CHI3L1)* and Leucine Rich Repeat Containing G Protein-Coupled Receptor 6 *(LGR6)* genes were located in the top ranked region on CFA7 (0.02–1 Mb) in the Qinghai-Tibetan Plateau dogs. Both genes have been associated with hypoxia responses or altitude adaptation in humans. For the Xi dogs, the top ranked region on CFA25 contained the Transient Receptor Potential Cation Channel Subfamily C Member 4 *(TRPC4)* gene. This calcium channel is important for optimal muscle performance during exercise. The outstanding signals in the Mountain dogs were on CFA5 with 213 significant SNPs that spanned genes involved in cardiac development, sight and generation of biochemical energy. These findings support the use of the combined index approach for identifying novel regions of genome diversity in dogs. As with other methods, the results do not prove causal links between these regions and phenotypes, but they may assist in focusing future studies that seek to identify functional pathways that contribute to breed diversity.

## Introduction

The development of modern dog breeds from ancestral populations is a good model for understanding domestication and genetic diversification^1^. The first domestication events created genetic bottlenecks that have since been exacerbated by further selective pressures in the creation of specific breeds. Around 500 distinct breeds are currently recognized by breed clubs, such as the American Kennel Club (https://www.akc.org/)^2,3^, Australian National Kennel Council (https://dogsaustralia.org.au/)4,5 or The Kennel Club (UK, https://www.thekennelclub.org.uk/)^6,7^. Each breed is classified by a standard, which includes morphological criteria, behavioural traits and coat color^8,9^. These guidelines were applied during the development of modern pure-breed dogs, consequently phenotypic and genetic heterogeneity has been substantially reduced within breeds while maintaining diversity across breeds.

Previous studies of genomic diversity in dogs have used summary statistics to measure locus specific divergence in allele frequencies, for example *F*_*st*_, XP-EHH and *di*^10–15^. Akey et al.^12^ demonstrated that, combined with high density SNP markers, the *F*_*st*_ statistic was a powerful tool to scan the canine genome for selection signatures, and developed a modified pairwise statistic (*di*) based on *F*_st_ to detect locus specific deviation between breeds ^10^. The other widely used statistical test to identify regions of interest is Cross Population EHH (XP-EHH)^16,17^. Using long-range haplotype information, XP-EHH measures whether a selected allele has risen in frequency in one population but not in a second reference population^16^. The use of a single methodology may be enhanced by combining signals across different tests^18–20^. One such composite method, named composite selection signals (CSS), has been developed in domesticate species (cattle and sheep) for application to genomic data where a detailed individual phenotypic or population history is not available^21,22^. Application of the CSS method to cattle breeds and across species (cattle and sheep) have detected signals throughout the genome with a high degree of sensitivity.

The aim of this study was to investigate the utility of the CSS method for exploring diversity in three groups of geographically distinct Asian dogs: Qinghai-Tibetan Plateau dogs, Shandong and Shaanxi Xi dogs, and Sichuan Mountain hounds ^11^.

## Results

Three groups of dogs were investigated individually as the targets of analysis, similar to Yang et al.^11^: Qinghai-Tibetan Plateau dogs , Xi dogs and Mountain hounds. Dogs that reside in the high Qinghai-Tibetan Plateau have adapted to life at high altitudes. The other two dog groups are geographically distinct and are generally described as having superior running and hunting ability, respectively, but morphological and physiological traits in the Xi and Mountain hound breed groups are not specifically defined.

**Figure 1:**
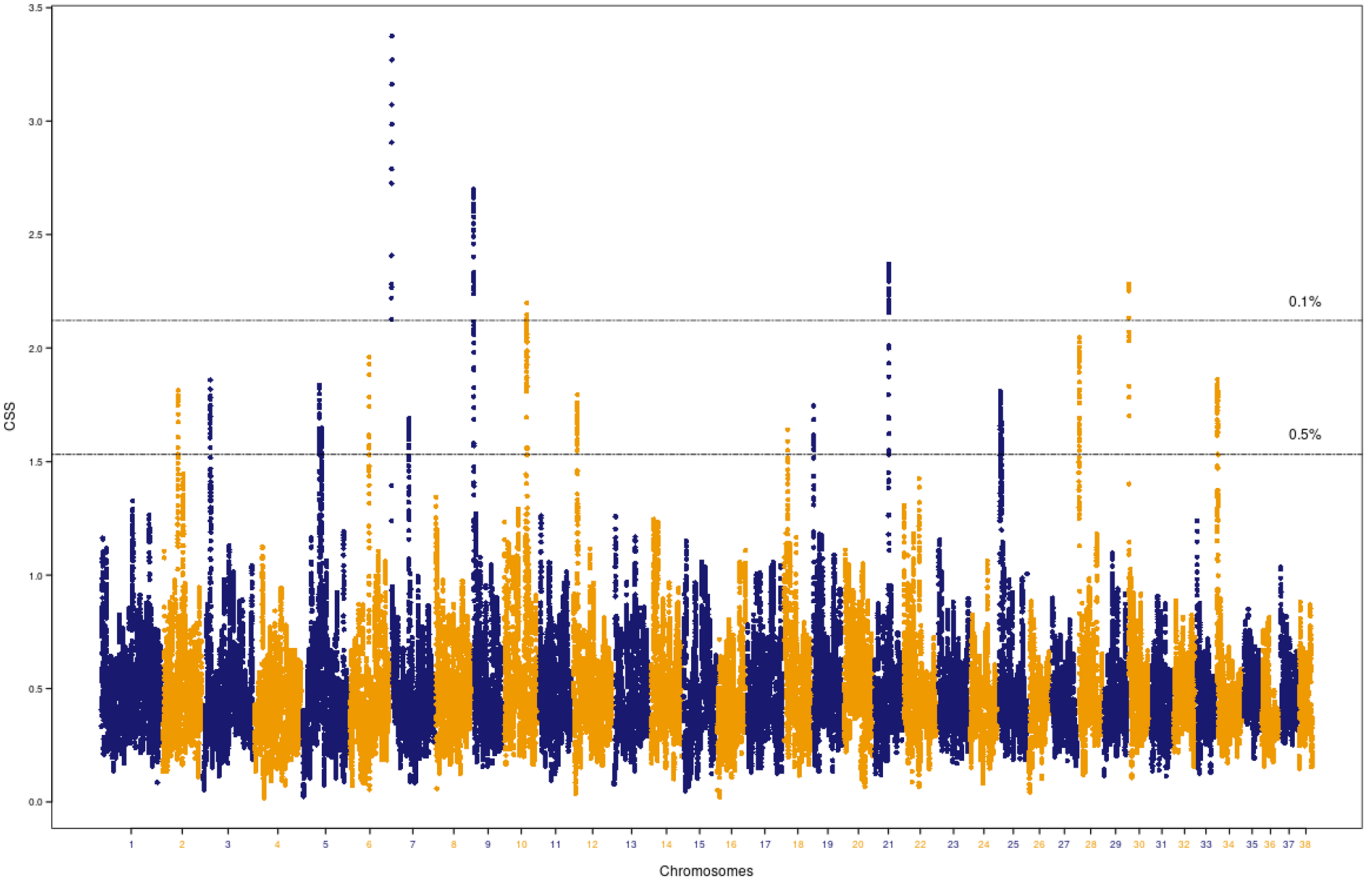
Composite selection signals associated with adaptation to high altitude. CSS statistics were determined with Qinghai-Tibetan Plateau dogs as the target group. The canine chromosomal positions of SNP markers are plotted on the x-axis. The observed values for selection scan statistics based on the combination of F*st*, ∆DAF and XP-EHH are highlighted in blue and orange. Each point represents the smooth CSS value in a 1 Mb overlapping sliding window across the autosomes. The two-dashed horizontal lines indicate threshold values for the top 0.1% (top) or 0.5% (bottom) of CSS values.

Living at high-altitude in the Himalayan Mountains resulted in physiological adaptations that allowed dogs to perform in low oxygen conditions. Hequ Tibetan Mastiffs and Tibetan Mastiffs are ancient breeds of large dogs, native to the northeastern part of the Qinghai-Tibetan Plateau of China and Linzhi, located in the southeastern part of the Tibet Autonomous Region (TAR) of China. They adapted to a high-altitude environment over a relatively short period of time. A genome-wide scan using these dogs as the target group and a 1 Mb sliding window size identified a total of 1,397 significant SNPs (Table 1). Based on the smoothed CSS value (3.38), the top SNP was on CFA7 (CFA7: 1,000,539). Details of the analysis for all the significant SNPs is provided in Supplementary Table S1. When the threshold for significant regions was applied, these SNPs clustered into 17 genomic regions (Fig. 1), defined by the first and last significant SNP in the window. The 17 regions were located on 15 different chromosomes including CFA2, CFA3, CFA5, CFA6, CFA7, CFA9, CFA10, CFA12, CFA18, CFA19, CFA21, CFA25, CFA28, CFA30, and CFA34 (Table 2). The top region based on the average of the smoothed CSS score (2.83) for 37 SNPs across the region was on CFA7 (0.02–1 Mb).

**Table 1:** Summary of the significant SNPs from CSS analysis.

Phenotype	Total No. of SNP	Window size	Threshold (%)	No. of regions	Significant SNPs
Qinghai- Tibetan dogs adapted to altitude	123,112	200 Kb	0.1	17	246
			0.5	58	1,319
		1 Mb	0.1	5	299
			0.5	17	1,397
Xi dogs with superior running capacity	123,100	200 Kb	0.1	19	250
			0.5	60	1,421
		1 Mb	0.1	19	250
			0.5	23	1,675
Mountain dogs with hunting ability	123,578	200 Kb	0.1	12	289
			0.5	53	1,237
		1 Mb	0.1	3	295
			0.5	17	1,475

**Table 2:** Selected genomic regions in Qinghai-Tibetan Plateau dogs adapted to high altitude.

CFA	Region	Range of smoothed CSS values	Av. smoothed CSS value	No. of SNPs	Significant SNPs
2	34.71–35.41 Mb	1.13–1.81	1.69	52	48
3	14.49–15.74 Mb	0.8–1.86	1.73	86	79
5	30.85–32.51 Mb	0.65–1.84	1.62	99	95
	35.1–36.51 Mb	1.15–1.52	1.61	96	89
6	36.84–38.38 Mb	0.56–1.96	1.69	69	59
7	0.02–1 Mb	0.93–3.37	2.83	37	37
	33.95–35.22 Mb	0.55–1.69	1.62	75	68
9	0.01–1.47 Mb	1.41–2.7	2.28	87	84
10	47.68–49.6 Mb	0.71–2.2	1.96	126	117
12	6.55–8.02 Mb	0.72–1.79	1.66	82	82
18	11.11–12.28 Mb	0.28–1.64	1.59	46	46
19	0.33–1.72 Mb	0.82–1.74	1.61	40	40
21	27.24–29.17 Mb	0.45–2.24	2.11	127	117
25	2.58–6.51 Mb	0.76–1.81	1.65	196	195
28	0.03–1.82 Mb	0.64–2.03	1.83	91	91
30	0–1.02 Mb	0.65–2.28	2.15	37	37
34	0.59–2.3 Mb	0.73–1.86	1.74	113	113

**Figure 2:**
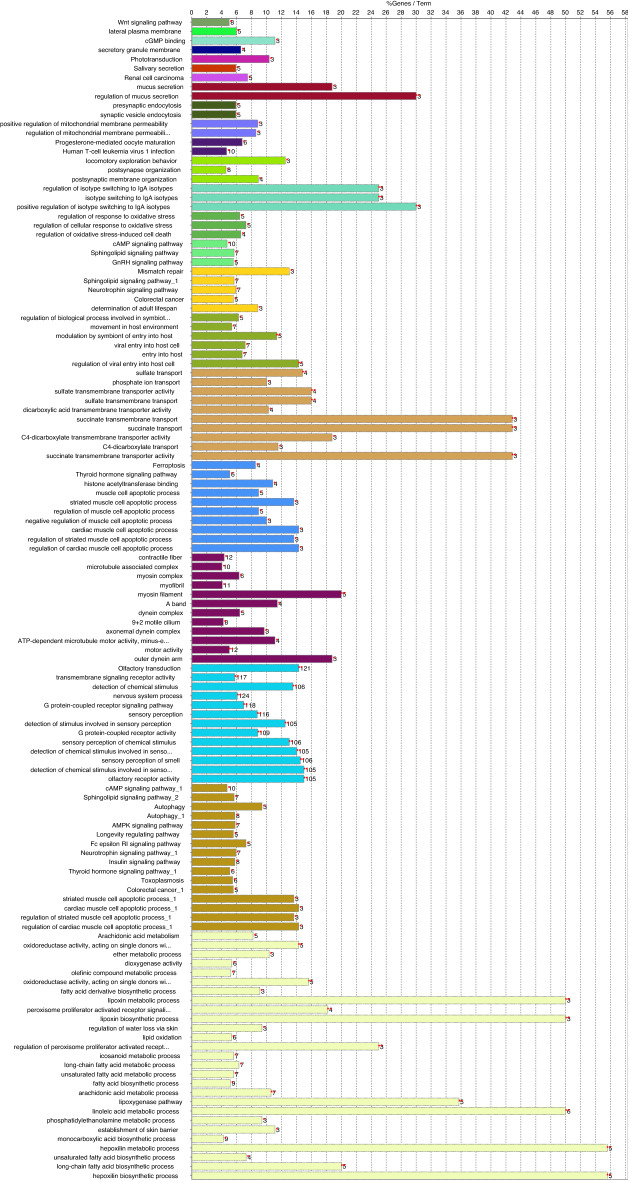
Functional annotation groups for genes associated with adaptation to high altitude. See Table S4 for details.

When genomic regions identified in the Qinghai-Tibetan Plateau dog population were examined, they contained a total of 220 annotated genes (ROS CFam v1.0). This list of genes was subjected to gene set analysis. The findings in each individual gene ontology category (biological process; molecular function; and cellular component) or pathway analysis that satisfied cut-off criteria (Benjamini < 0.05 or FDR < 0.05), are listed in Supplementary Table S2. The top enrichment clusters are listed in Supplementary Table S3. The top enrichment clusters included GO terms linked to molecular functions associated with aerobic metabolism, response to oxygen levels and hypoxia. Other prominent clusters linked to the biological processes of lung development and muscle function. Similar results were generated when the ClueGO v2.2.8/CluePedia v1.5.8 tools were employed (Fig. 2). The genes and flanking neighbors were classified into 23 groups, with the most significant terms of each group shown in Supplementary Table S4. The detection of genomic regions containing genes and significant pathways linked to oxygen availability and known to be associated with physiological adaptation to high altitude, demonstrates the capacity of the CSS method to identify distinctive genome diversity in the Qinghai-Tibetan Plateau dog breeds.

**Figure 3:**
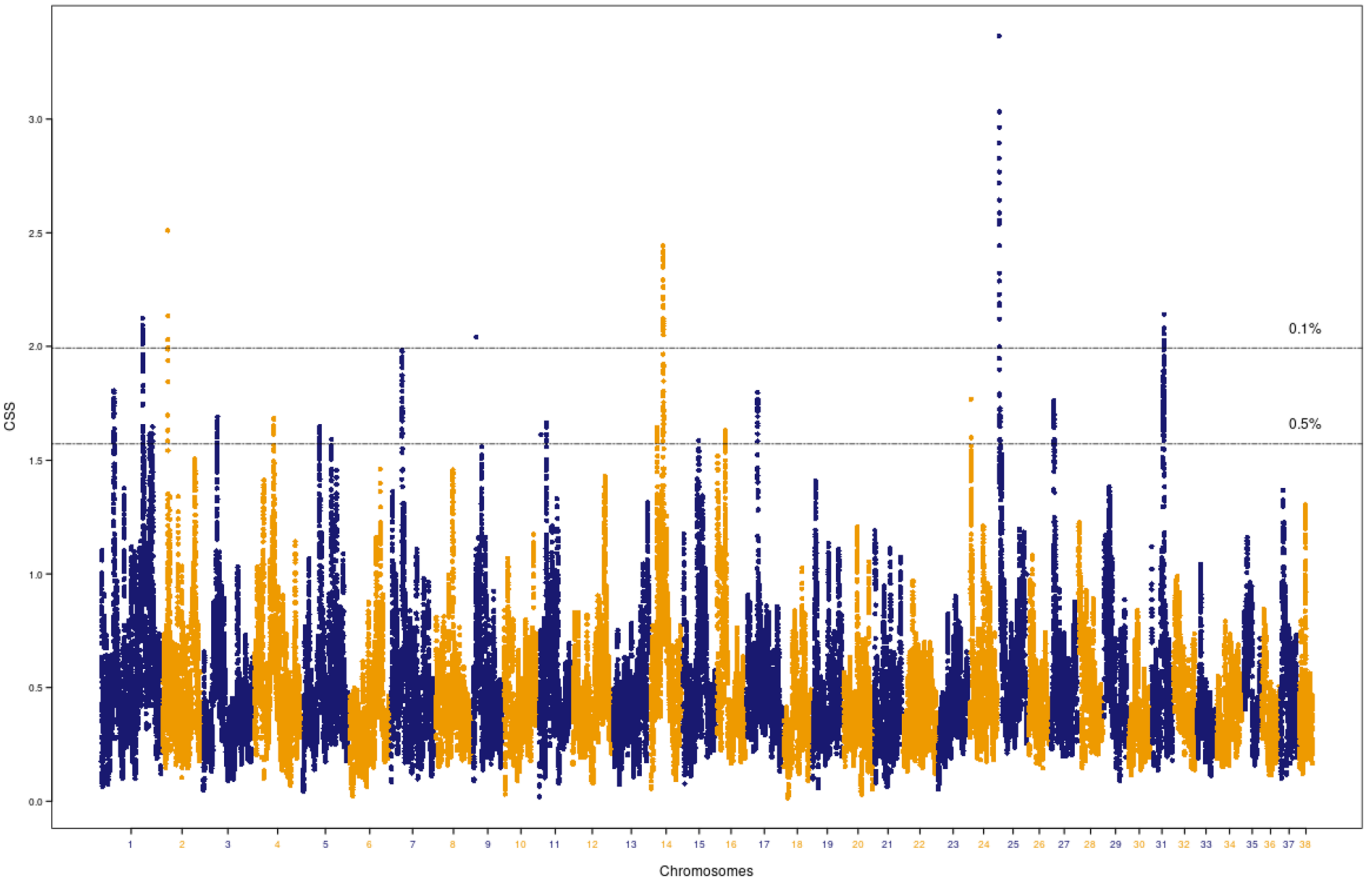
Composite selection signals associated with running speed. CSS statistics were determined with Xi dogs as the target group. The canine chromosomal positions of SNP markers are plotted on the x-axis. The observed values for selection scan statistics based on the combination of F*st*, ∆DAF and XP-EHH are highlighted in blue and orange. Each point represents the smooth CSS value in a 1 Mb overlapping sliding window across the autosomes. The two-dashed horizontal lines indicate threshold values for the top 0.1% (top) or 0.5% (bottom) of CSS values.

Shandong Xi dogs and Shaanxi Xi dogs are considered two of the ancient dog breeds in China. The long-haired Xi dog and short-haired Xi dog were once widely distributed in Hebei, Shandong, Shaanxi, Mongolia and other places. A genome-wide CSS scan for the Xi dogs identified a total of 1,475 significant SNPs (Table 1). Based on the smoothed CSS value (3.36), the top SNP was on CFA25 (CFA25: 1,538,793). Details of the analysis for all SNPs is provided in Supplementary Table S5. The top SNPs clustered into 23 genomic regions (Fig. 3). These regions were located on 16 different chromosomes, including CFA1, CFA2, CFA3, CFA4, CFA5, CFA7, CFA9, CFA11, CFA14, CFA15, CFA16, CFA17, CFA24, CFA25, CFA27 and CFA31 (Table 3). The top ranked region based on the average of smoothed CSS scores (2.18) for 129 SNPs was on CFA25 (1.54–3.85 Mb).

**Table 3:** Selected genomic regions in Xi dogs with superior running capacity.

CFA	Region	Range of smoothed CSS values	Av. smoothed CSS value	No. of SNPs	Significant SNPs
1	24.21–26.16 Mb	0.86–1.81	1.69	114	107
	80.57–82.43 Mb	0.9–2.12	1.87	110	99
	92.59–93.68 Mb	0.91–1.61	1.60	60	53
	95.27–96.26 Mb	0.98–1.6	1.60	57	50
	99.38–100.34 Mb	1.02–1.65	1.61	50	41
2	12.75–14.39 Mb	0.84–2.53	1.90	58	50
3	30.05–31.68 Mb	0.72–1.69	1.62	82	75
4	38.08–39.21 Mb	1.19–1.68	1.64	71	64
5	31.94–33.43 Mb	1.1–1.65	1.60	78	74
	56.66–57.65 Mb	1.16–1.59	1.59	49	45
7	20.6–22.22 Mb	0.46–1.99	1.79	79	79
9	8.03–8.38 Mb	2.04	2.04	21	19
11	10.12–10.18 Mb	1.61	1.61	3	3
	21.77–22.91 Mb	1.03–1.66	1.62	64	64
14	14.72–16.18 Mb	0.78–1.64	1.61	96	92
	25.86–27.93 Mb	0.93–2.44	2.03	129	121
15	34.28–34.75 Mb	1.4–1.59	1.59	26	24
16	22.99–24.31 Mb	1.11–1.63	1.60	73	72
17	18.66–20.1 Mb	0.5–1.77	1.72	75	62
24	2.81–4.08 Mb	1.21–1.77	1.71	46	46
25	1.54–3.85 Mb	1.35–3.37	2.18	130	129
27	3.15–4.97 Mb	0.59–1.76	1.67	105	105
31	20.73–24.23 Mb	0.69–2.03	1.80	226	210

**Figure 4:**
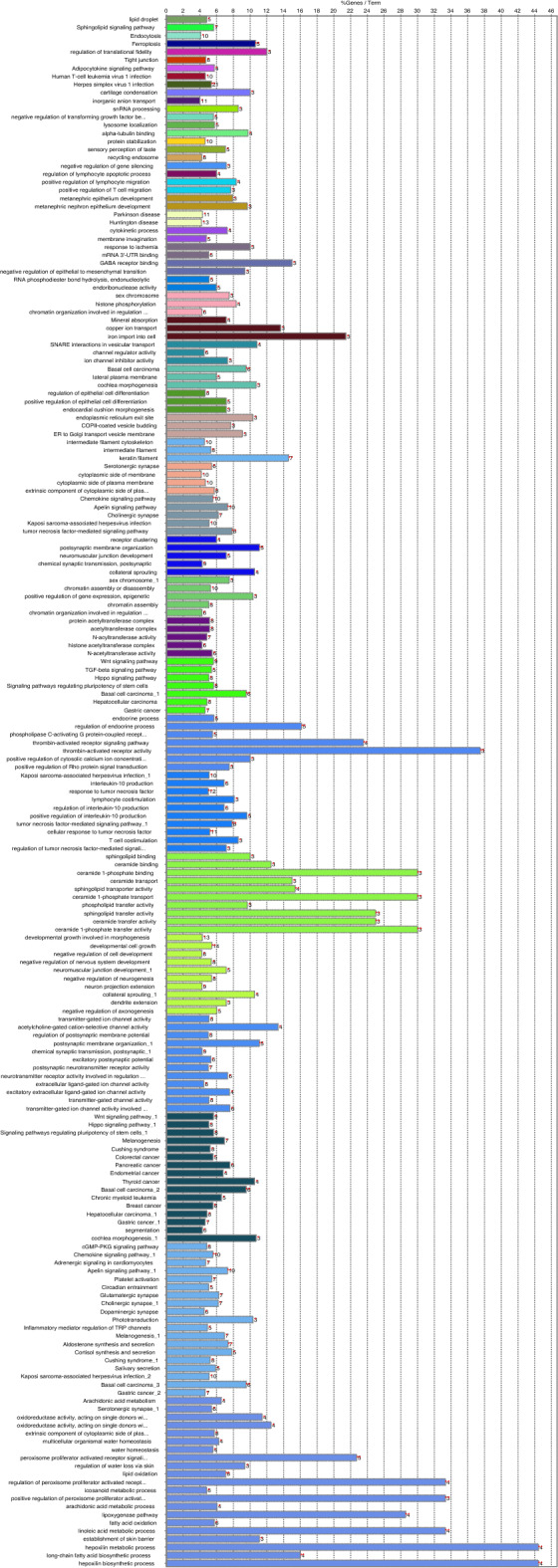
Functional annotation groups for genes associated with running speed. See Table S8 for details.

The 23 significant genomic regions contained 242 annotated genes. The full lists of GO term clusters and enrichment analysis is provided in Supplementary Tables S6 and S7. In the most significant enrichment cluster the GO terms were the response to regulation of transcription and acetylcholine activated ion channel activity. Others were linked to the biological processes of systemic arterial blood pressure, limb morphogenesis and development, and heart process. In order to understand the most significant grouped annotations ClueGO v2.2.8/CluePedia v1.5.8 was employed to create functional clusters (Fig. 4). Details of significant terms of each group are shown in Supplementary Table S8. Amongst these genes, we identified enrichment in genes involved in cardiovascular function and acetylcholine-gated cation-selective channel activity, both may contribute to superior running ability in Xi dogs^15,23^. Indeed, the efficient operation of cation channels at neuromuscular junctions is critical for skeletal muscle contraction during running^24–26^.

**Figure 5:**
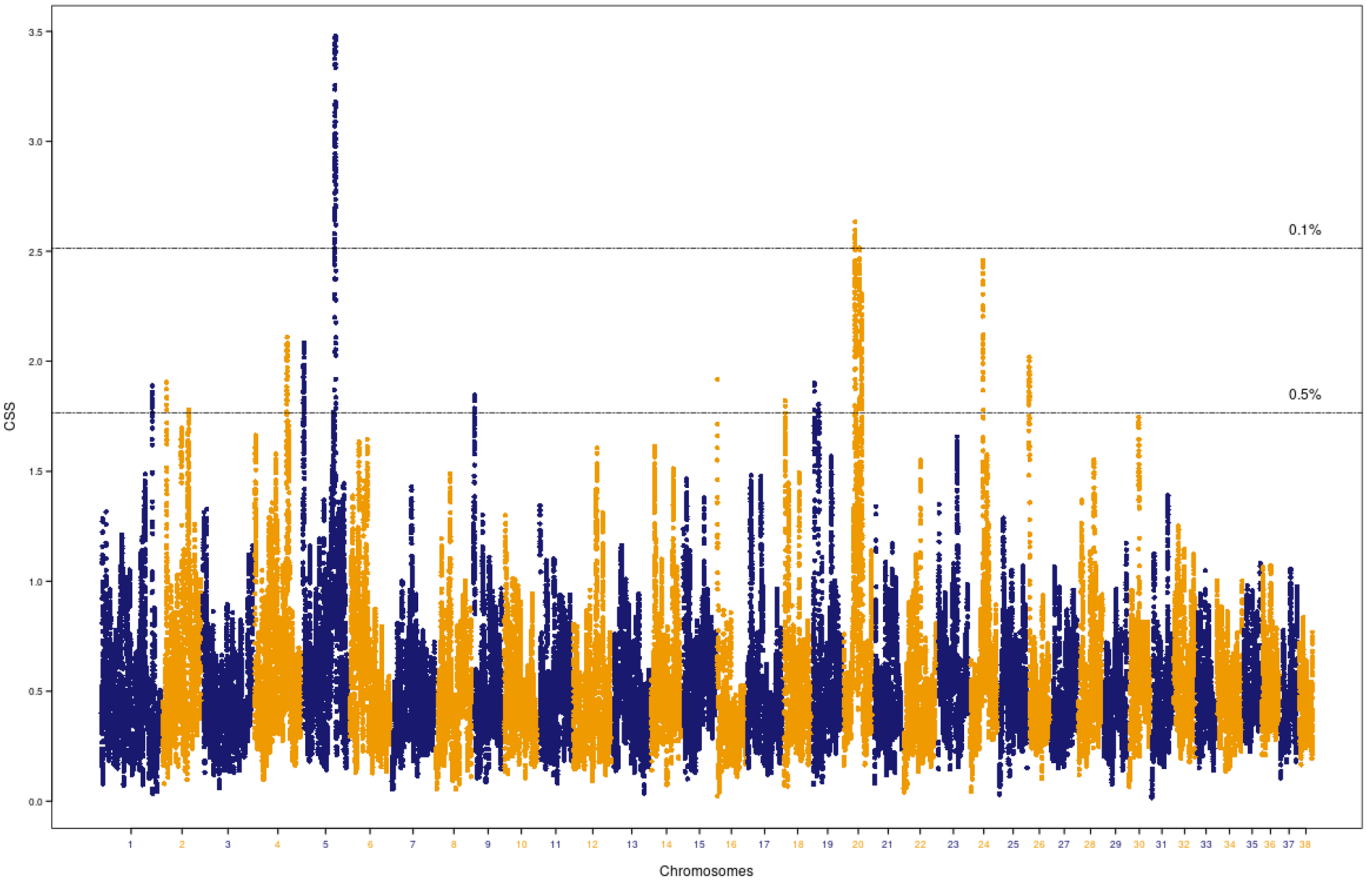
Composite selection signals associated with hunting ability. CSS statistics were determined with Mountain hounds as the target group. The canine chromosomal positions of SNP markers are plotted on the x-axis. The observed values for selection scan statistics based on the combination of F*st*, ∆DAF and XP-EHH are highlighted in blue and orange. Each point represents the smooth CSS value in a 1 Mb overlapping sliding window across the autosomes. The two-dashed horizontal lines indicate threshold values for the top 0.1% (top) or 0.5% (bottom) of CSS values.

Liangshan and Qingchuan Mountain hounds are Chinese native breeds mainly distributed in a landlocked mountainous region of Western Sichuan province. They were traditionally used for hunting. These dogs were compared to a group of geographically distinct breeds. A genome-wide CSS scan using the Mountain hounds as the target group identified a total of 1,675 significant SNPs (Table 1). Based on the smoothed CSS value (3.48), the top SNP was located on CFA5 (CFA5: 64,218,702). Details of the analysis for all SNPs is provided in Supplementary Table S9. The top SNPs clustered into 17 genomic regions (Fig. 5). The regions were located on 11 different chromosomes, including CFA1, CFA2, CFA4, CFA5, CFA9, CFA16, CFA18, CFA19, CFA20, CFA24 and CFA26 (Table 4). The top ranked region based on the average of smoothed CSS scores (2.79) of 213 SNPs was on CFA5 (62.08–65.6 Mb).

**Table 4:** Selected genomic regions in Mountain hounds used for hunting.

CFA	Region	Range of smoothed CSS values	Av. smoothed CSS value	No. of SNPs	Significant SNPs
1	98.23–99.38 Mb	0.71–1.89	1.82	57	51
2	10.8–11.9 Mb	0.88–1.9	1.84	69	63
	62.23–63.24 Mb	1.06–1.78	1.78	59	59
4	64.03–65.57 Mb	1.10–2.14	1.97	95	81
5	1.05–3.92 Mb	1.0–2.08	1.91	125	125
	59.41–60.4 Mb	1.44–1.71	1.77	76	68
	62.08–65.6 Mb	0.5–3.48	2.79	223	213
9	1.58–2.79 Mb	1.21–1.84	1.80	46	46
16	2.61–3.05 Mb	1.15–1.92	1.92	22	22
18	6.01–7.04 Mb	0.93–1.82	1.80	73	73
19	3.41–4.57 Mb	0.64–1.9	1.88	45	45
	12.88–14.03 Mb	0.87–1.81	1.79	63	60
20	20.65–23.09 Mb	1.07–2.63	2.19	164	159
	28.52–30.82 Mb	1.12–2.51	2.24	126	116
	32.88–35.17 Mb	1.08–2.30	2.04	145	127
24	22.48–24.14 Mb	0.71–2.46	2.17	91	84
26	0.01–1.44 Mb	0.83–1.91	1.89	83	83

In the Mountain hound population, the 17 significant genomic regions contained 198 annotated genes. The list of GO term annotations and pathway descriptions is provided in Supplementary Table S10. When the genes were subjected to GO term cluster enrichment analysis and KEGG pathway analysis with neighboring genes, the top enrichment cluster was linked to the response to regulation of transcription, DNA-templated, nucleic acid-templated transcription and RNA biosynthetic process. Others corresponded to the response to photoreceptor cell development, differentiation and retina development (Supplementary Table S11).

**Figure 6:**
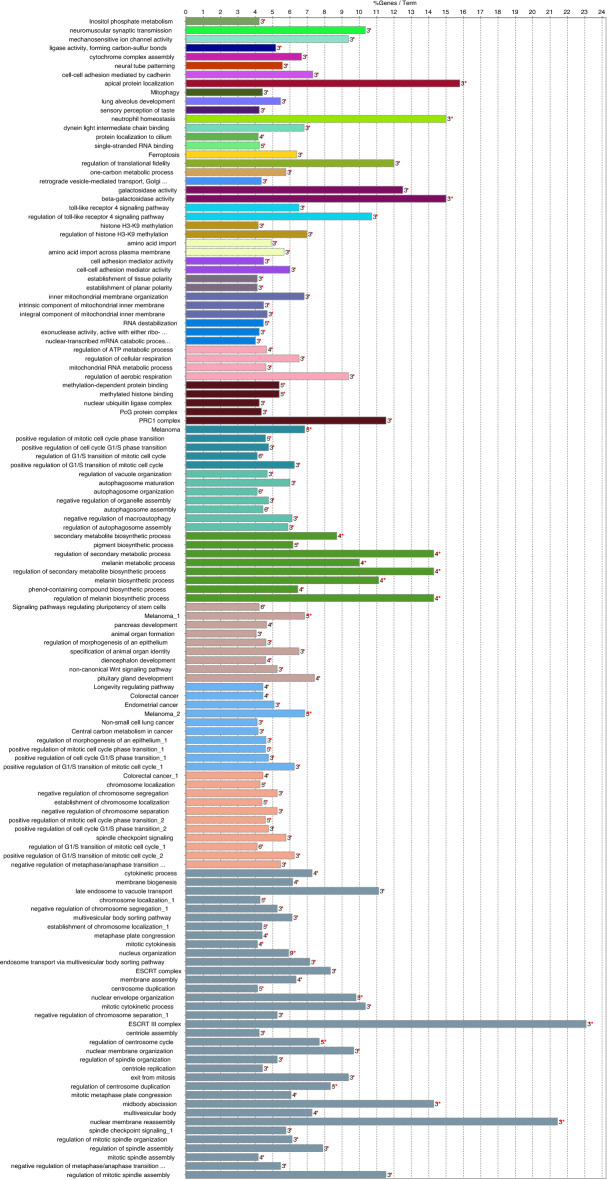
Functional annotation groups for genes associated with hunting ability. See Table S12 for details.

Similar to the previous sections, to present the relevant grouped annotation networks, ClueGO v2.2.8/CluePedia v1.5.8 was employed. These groups are shown in Fig. 6. The most significant terms for each group are presented in Supplementary Table S12. The pathways and networks in this analysis related to broad biological processes. This may reflect the complex nature of hunting ability in dogs and the difficulty in precisely defining the trait. However, the outstanding signal on CFA5 corresponded to an interesting region containing genes involved in vision, cardiac development, metabolism and mitochondrial function. This is consistent with superior hunting ability in which a keen sense of sight, and an elevated aerobic capacity for sustained tracking, may be highly developed.

## Discussion

The availability of genotype data for distinct groups of Asian dogs provided an opportunity to explore the application of the CSS method. By combining multiple tests (*Fst*, ΔDAF/SAF, and XP-EHH) this analysis confirmed regions described in similar studies and identified novel distinctive regions in each of these breed groups. Similar results were generated when the SAF method was substituted for DAF and is most useful in circumstances where ancestral allele determination is not possible (Supplementary Figures S1–S3).

Dogs and other species that have adapted to life at high altitudes have been the subject of several studies^11,27–29^. These studies provided the most detailed data for comparison with the present study. Physiological adaptation to altitude includes the response to low oxygen concentrations and subsequent cell hypoxia, and control of inflammatory responses that may affect cardiovascular smooth muscle and capillary permeability, with consequences for normal lung and brain function. Hypoxia inducible factors (HIFs) play an important role in regulating external and internal adaptation to hypoxic stress^30,31^, while inflammatory pathways are characterized by signaling through the effector molecule, Nuclear Factor kappa B (NF-$\kappa$B)^32^. Proteasomal degradation is an important regulatory mechanism in both pathways^33^. Although breed-specific haplotype variants cannot infer functional relationships, variants surrounding genes involved in these pathways have featured in genome analyses of altitude adaptation in a range of species and are prominent in the regions identified in the present study.

In other canine studies, selection signals have been detected using single methods or by analysis using two methods independently to verify findings. When our results were compared to the study used as the source of genotype data, two regions in common were found on chromosome 10 and 21, both in the top 0.1% of CSS signals in our study. The CFA10 region contains the gene for the HIF regulatory protein *EPAS1* which has been recognized in previous analyses of Tibetan Mastiffs^34,35^ and has been shown to contain non-synonymous variants in these dogs. Another mediator of hypoxia signaling pathways, *PRKCE* (protein kinase C epsilon), was also found in the CFA10 region, while the key regulator of HIF pathways, *P4HB* (prolyl 4-hydroxylase beta polypeptide)^30,31^, was within a significant region on CFA9.

The common region identified on CFA21 in both studies is rich in olfactory receptor genes with *OR52A1* originally named as a possible candidate. The olfactory receptor gene family is the largest gene family in the genome and is particularly rich in dogs. However, this can be confounding when prioritizing gene candidates. We note that embedded within the olfactory receptor genes in this region is the dog hemoglobin beta chain gene (*HBB)* and suggest that this is the likely candidate for these signals, similar to Fan et al*.*^34^. *HBB* has been previously recognized in other species as a gene under selection in response to altitude adaptation^36^. Surprisingly, the *HBB* gene is only provisionally annotated in the dog genome assembly as *LOC609402*, and this may have resulted in this gene being overlooked as a candidate in some studies.

The significant region identified here on CFA5 from the analysis of Qinghai-Tibetan Plateau dogs contains a number of genes that may contribute to altitude adaptation. Dynein Axonemal Heavy Chain 9 *(DNAH9)* was named by Yang et al*.*^11^, and Kinesin Family Member 1C *(KIF1C)* and Enolase 3 (*ENO3)* were added to the potential candidates in this location by Li et al*.*^35^. One other gene at this location is Myocardin (*MYOCD*) which have never been mentioned in the context of dogs, but we note is associated with altitude adaptation in humans^37^. We also identified genes with functions related to altitude adaptation in a region on CFA28. For example, Arachidonate Lipoxygenase gene family member 5 *(ALOX5)* which has a role in oxidoreductase activity, oxygen and iron binding, is potentially a novel candidate for these dogs^38^. Two other genes, Kinesin Family Member 6 *(KIF6)* and ERCC Excision Repair 6 *(ERCC6)*, were also previously reported as candidate genes^35^.

Chromosome 7 had the highest CSS value in our study but has not been picked up by previous studies on Tibetan Mastiffs. This region includes the Chitinase 3 Like 1 *(CHI3L1)* and Leucine Rich Repeat Containing G Protein-Coupled Receptor 6 *(LGR6)* genes. *CHI3L1* has been identified in altitude induced pulmonary oedema and the response of lung tissue to inflammation in humans^39,40^. *LGR6* is important for proliferation of lung epithelial cell progenitors to maintain tissue homeostasis and normal lung function^41^.

Other regions identified corresponded to those found in Tibetan Mastiffs by Li et al*.*^35^ and included a region on CFA9 with the potential candidate sodium-coupled neutral amino acid transporter 10 *(SLC38A10)*. Both studies also identified a region on CFA30 where the calcium ion release channel Ryanodine Receptor 3 *(RY3R)* gene is located, and CFA25 containing the Doublecortin Like Kinase 1 *(DCLK1)* gene.

Uniquely identified regions included a region on CFA34 containing *OTULIN*, a deubiquitinase that affects signaling via Mammalian Target of Rapamycin (mTOR) and Nuclear Factor kappaB (NF-$\kappa$B), and is essential for restraining spontaneous inflammatory responses in the lungs^42^, which may be induced by altitude in naive animals. An additional region on CFA6 contained Vasorin *(VASN)*, which has been found to influence the response to hypoxia and angiogenesis, and Polycystin-1 (PKD1) which has been shown to influence lung development and inflammatory responses. The Rieske Fe-S domain containing *(RFESD)* gene was found in a significant region on CFA3. This gene encodes a molecule with iron and oxygen binding functions.

Running speed and strength are dependent on musculoskeletal and neuromuscular physiological processes involving balance, coordination, neuromuscular function and muscle development (myogenesis). The regions identified in the analysis of Xi dogs included genes that are central to these processes, most notably those involved in cerebellar function, excitation–contraction coupling in muscle and muscle development. The top ranked region on CFA25 contained the Transient Receptor Potential Cation Channel Subfamily C Member 4 *(TRPC4)* gene, which codes for a protein that, together with *TRPC1* and stromal-interacting molecule-1 *(STIM1)*, is an important regulator of a process known as store-operated calcium entry (SOCE)^43,44^. This process is critical for optimal skeletal muscle function during exercise when there are repetitive sarcolemma membrane depolarizations. SOCE has been implicated in fatigue resistance and in the development and maintenance of muscle mass. Another of the CSS identified selected regions on CFA1 overlapped with that described by Yang et al*.*^11^ and contained *NOL8* and *IARS*, but the region they highlighted on CFA9 containing multiple keratin genes was not significant in our study.

Other genes in CSS top ranked selected regions were associated with myofiber development and function. Notable amongst the top annotations of genes within the identified selected regions were those associated with acetylcholine receptor function. Acetylcholine is the neurotransmitter found at the neuromuscular junction and initiates muscle contraction, a key point for continuous skeletal muscle function during exercise in dogs^25^.

The analysis of Mountain hounds highlighted multiple signals on CFA5 and CFA20, followed by CFA24. The top ranked region on CFA5 (62.08–65.6 Mb) corresponded to a region identified by Yang et al*.*^11^ and contained genes involved in eye development and sight (*RBP7* and *NPHP4*), cardiac development *(UBE4B)* and regulation of metabolism *(H6PD).* An additional gene in this region, Spastic Paraplegia 7 *(SPG7),* affects mitochondrial function. Variants in *SFG7* in humans are known to affect muscle ATP production, eye movement and Purkinje neurons of the cerebellum, critical for motor coordination and balance^45,46^.

Numerous windows-based scan methods have been utilized for detecting signatures of selection in dogs and other species^10,14,21,22,47^. However, the number of adjacent SNPs included in a window is a variable parameter which requires optimization^48–50^. The ideal window size will differ depending on SNP array density, the pattern of linkage disequilibrium (LD) throughout the genome, the statistical methods applied and the genetic architecture of trait variation^51^. A comparison of two window sizes during analyses in this study showed that the size of the window had some influence on the final results, as previously noted^52^. However, LD (0.1 < r^2^ < 0.2) extends up to 0.3 Mb^11^ in the Chinese dog breeds, so the smaller 200 Kb window size may lose SNP information, whereas 1 Mb window size was considered more robust for this analysis and the addition of the smoothing steps within analysis windows has the capacity to incorporate the power of LD and reduce false positive signals due to single variant effects. Hence, a 200 kb window may add specificity to the analysis, but at the expense of sensitivity.

In this study we have applied the composite index method, CSS, to evaluate genomic regions that differentiate dogs from three breed groups. The CSS method has been shown to be a sensitive method for detecting selection signals in cattle and sheep, and here we have demonstrated its utility in canine sub-populations. Using an index approach improved sensitivity and robustness of identifying signals. A comparison to the relevant study by Yang et al*.*^11^ showed that the CSS method using the parameters specified here focused the significant regions in each analysis by reducing the number by approximately 50% while simultaneously increasing the number of potential candidate genes for each of the regions. It is important to note that these methods do not differentiate the nature of the variation which may arise from regions of selection, genetic drift or carrier haplotype mechanisms that may be affected by defining characteristics of the dogs under study^53^. However, some functional relevance of the candidate genes within the significant genomic regions was supported by in silico functional analysis. The impact of the identified gene networks and pathways on the respective breeds awaits further analysis, but interpretation would be improved if traits were quantified. Ultimately no causal link can be established without detailed functional studies. Such studies will benefit from the current application of the CSS method to defining breed differences in these dogs.

## Materials and Methods

### Data preparation

This study used a dataset containing genotypes from 167 samples from China. We defined four groups based on the previously published source genotype data^11^. The three geographically distinct dog groups: Qinghai-Tibet plateau dogs (*n* = 34), consisting of 15 samples of Tibetan Mastiffs, 7 samples of Hequ Tibetan Mastiffs and 12 samples of Linzhi dogs; Xi dogs (*n* = 24), consisting of 12 samples of Shandong Xi dogs and 12 samples of Shaanxi Xi dogs and merged as a target group with fast running speed (60 km/h); Mountain hounds (*n* = 24); consisting of 12 samples of Liangshan dogs and 12 samples of Qingchuan hounds, classified based on exceptional hunting ability. The fourth group is the reference groups and contains 85 samples including non-target dogs and additional samples from other Asian breeds (detail described in Supplementary Material Table S13). The original data was generated using the 170 K CanineHD BeadChip (Illumina, Inc., San Diego, CA) and was downloaded as filtered PLINK binary files from the Dryad data repository at https://datadryad.org/stash/share/u4FKRNZ4wueHyQEnYTeZ59XAWvuVg-aFMhTwqr1gfB4. A total of 151,057 SNP markers passed the quality control using PLINK 1.9^54^ with –maf 0.05 and –geno 0.1. The data was extracted into three groups representing dogs that were traditionally geographically distinct and noted for varied traditional utility (summarized in Supplementary Material Table S13).

### Phasing and haplotype

The SNP array data yielded unphased genotypes, making it difficult to observe whether different alleles were on the same haplotype. Hence, the BEAGLE 5.0 software^55,56^ (https://faculty.washington.edu/browning/beagle/beagle.html), was applied to assign haplotype phase from unphased genotype data. BEAGLE provides a population-based analysis with fast process time and high accuracy for medium to large sample sizes^57^. BEAGLE works by sampling haplotypes using a Hidden Markov model and is well suited to non-human datasets^58^. Prior to phasing, genotypic data was split by chromosome and converted to VCF format using PLINK v1.9^54^. The VCF files were used as input to BEAGLE using default settings (burnin = 3, iterations = 12, phased-states = 280, sliding windows = 40, overlap = 2, and no err parameter). The resulting haplotype information was then used in XP-EHH calculations.

### Cross-population extended haplotype homozygosity (XP-EHH)

The XP-EHH test was used to find alleles with an increase in frequency to the point of fixation or near-fixation in the populations under investigation. The SELSCAN^59^ package was used for computing XP-EHH scores. The EHH values were estimated by comparing samples from two groups via a target and a reference group. A positive value indicates that selection was likely to have occurred in the target group, while a negative score indicated selection happened in the reference group.

### Fixation index (Fst) analysis

The fixation index (*Fst*) has been widely used to measure genetic differentiation in positive selection and directly displays the variance in allele frequency between two populations^60,61^. The *Fst* values were determined as the mean values of allele frequency (method-of-moments) between the groups. The basis of the formula is as follows^52,61,62^: (*H*_T_–*H*_S_)/*H*_T_. Here *H*_T_ is a total counting of heterozygosity in populations and *H*_S_ is based on the average of heterozygosity across subpopulations.

### Allele frequency

Two methods of allele frequency analysis were compared, the selected allele frequency (SAF) and derived allele frequency (DAF). Both can be used to detect high frequency alleles in the target group and are sensitive for distinguishing selected alleles^18^, but SAF is calculated without prior knowledge of ancestral alleles.

The selected allele frequency is based on the observed major allele frequency for the subpopulation, hence ∆SAF = SAF *target*—SAF *reference*. The use of DAF required assignment of ancestral alleles and their frequencies. In the present study, the major alleles in wolves were assigned as ancestral alleles, following the approach used previously^50,63^ and in comparison with data from Alaskan Huskies as in a prior study^15^. The major alleles (common variant) from this dataset were assigned as ancestral alleles. A total of 166,579 SNPs were assigned for analysis. The derived allele frequency difference was computed according to the following formula: ∆DAF = DAF *target*—DAF *reference*. The normal distribution of derived allele frequency was estimated and the ∆DAF values were transferred to Z scores (0,1).

### Calculation of CSS values

This index was developed by Randhawa and colleagues^21,22^ and depends on rank-based *p*-values. Briefly, the CSS statistic is calculated from univariate measures (F*st*, ∆DAF/SAF and XP-EHH) as follows: univariate statistics are first converted to fractional ranks between 0 to 1 by 1/(n + 1) to n/(n + 1), where n is the number of SNPs. Next, fractional ranks are converted to Z-values using the inverse normal cumulative distribution function (CDF). Thirdly, the mean Z-score is taken from all statistical tests at each SNP position and converted to a *p*-value using the normal N(0, m^−1^) distribution, where m is the number of univariate statistics. The logarithm of the *p*-value (-log_10_ of *p*-values) is equivalent to the CSS statistic.

A sliding window method was applied to reduce background noise and enhance robustness of detected signals from genomic regions^21,22,50^. The windows were shifted on SNP positions along the genome in either a 200 Kb or 1 Mb sliding window. Significance threshold was set based on the calculated mean of CSS values of all the SNPs within a window and corresponding to top 0.1% or top 0.5%. Regions generated from the analysis were defined by the first and last significant SNP in the corresponding window. Overlapping windows were merged into a single region bound by the first and last significant SNP after merging. The average CSS value was the mean of smoothed CSS values of all significant SNPs in the region.

### Analysis of regions

Genome annotation was performed based on genomic regions derived from CSS analysis. Annotated genes from the Ensembl gene annotation database (CanFam3.1) were retrieved by matching chromosome and position. All processes were performed using R software (https://ggvs-rstudio.vip.sydney.edu.au/auth-sign-in).

Genes from all significant genomic regions were used for functional enrichment analysis. The Database for Annotation, Visualisation and Integrated Discovery (DAVID) v6.8 was used for analyzing functional classification, gene ontology, interconnected pathways, and understanding high-level functions and biological systems from large-scale molecular datasets (http://david.abcc.ncifcrf.gov/)65,66 via Kyoto Encyclopedia of Genes and Genomes (KEGG, https://www.kegg.jp/ ) and Gene Ontology (GO, http://geneontology.org/ ) knowledge base resources. The Benjamini corrected *p*-value ≤ 0.05 and FDR ≤ 0.05 were used to indicate a statistically significant difference.

Additional analysis used the Cytoscape software (Version 3.8.2)^66^ plug-in ClueGO v2.2.8/CluePedia v1.5.8^67,68^ to investigate whether identified genes were biologically interconnected. Three independent ontologies, biological process (BP), molecular function (MF), cellular component (CC) categories, and one pathway analysis (KEGG) were constructed to grouped functional categories. The selection criteria applied the hypergeometric test (two-sided with *p* ≤ 0.05, Benjamini–Hochberg correction, and kappa score ≥ 0.4) and the significance was set as 0.05.

## Supplementary Information

Supplementary Information 1.

Supplementary Information 2.

Supplementary Information 3.

Supplementary Information 4.

Supplementary Information 5.

Supplementary Information 6.

Supplementary Information 7.

Supplementary Information 8.

Supplementary Information 9.

Supplementary Information 10.

Supplementary Information 11.

Supplementary Information 12.

Supplementary Information 13.

Supplementary Information 14.

## Data Availability

All data analysed during this study are included in this published article \cite^11^ and can be found in the Dryad database; https://datadryad.org/stash/share/u4FKRNZ4wueHyQEnYTeZ59XAWvuVg-aFMhTwqr1gfB4.
