# Supplementary material for: Analysis of dog breed diversity using a composite selection index
Source: Sci Rep. 2023 Jan 30;13:1674. doi: 10.1038/s41598-023-28826-3 (PMC9886904; doi:10.1038/s41598-023-28826-3)
Supplement: Supplementary file 1 — Supplementary Information 1. [file 41598_2023_28826_MOESM1_ESM.docx]

## Supplementary material


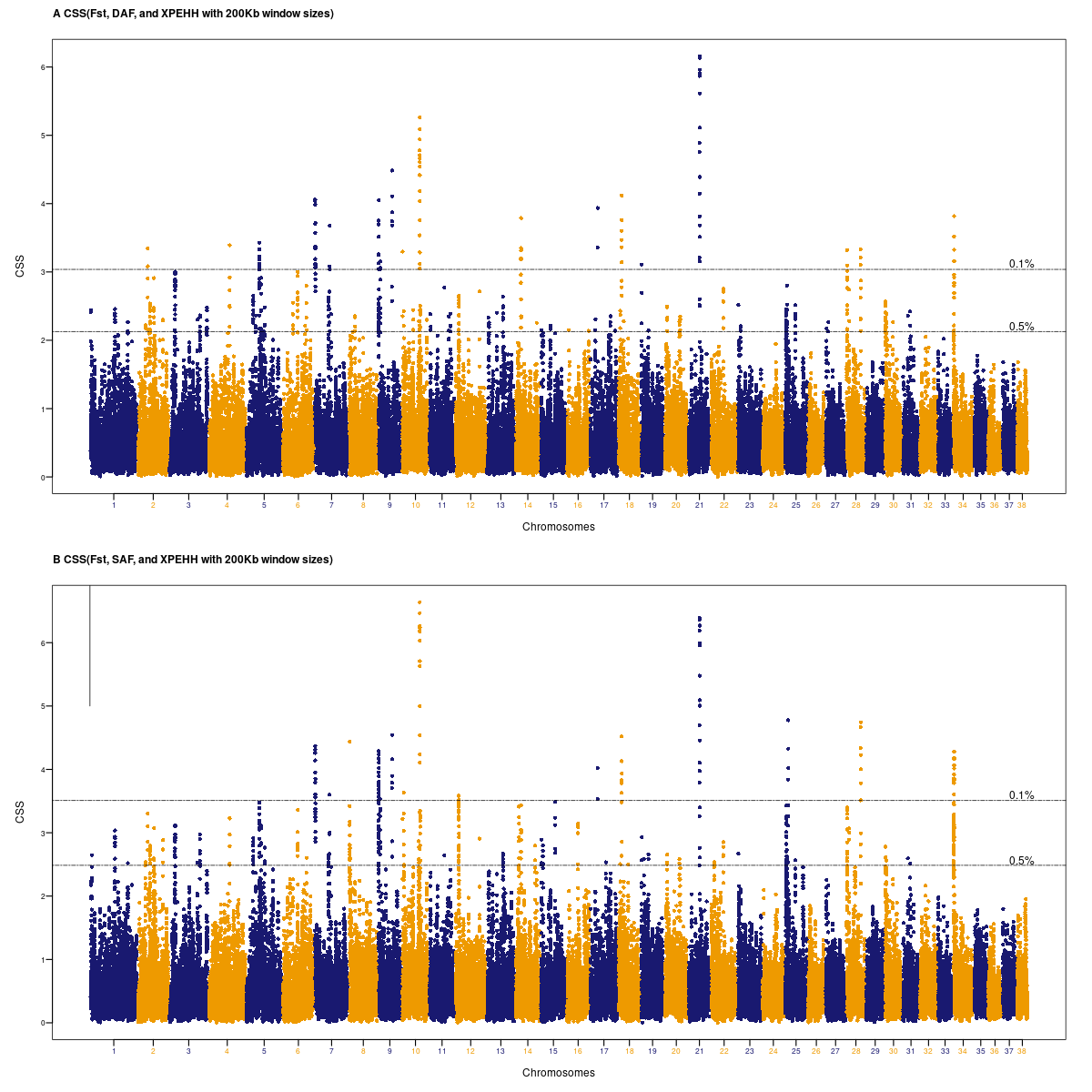


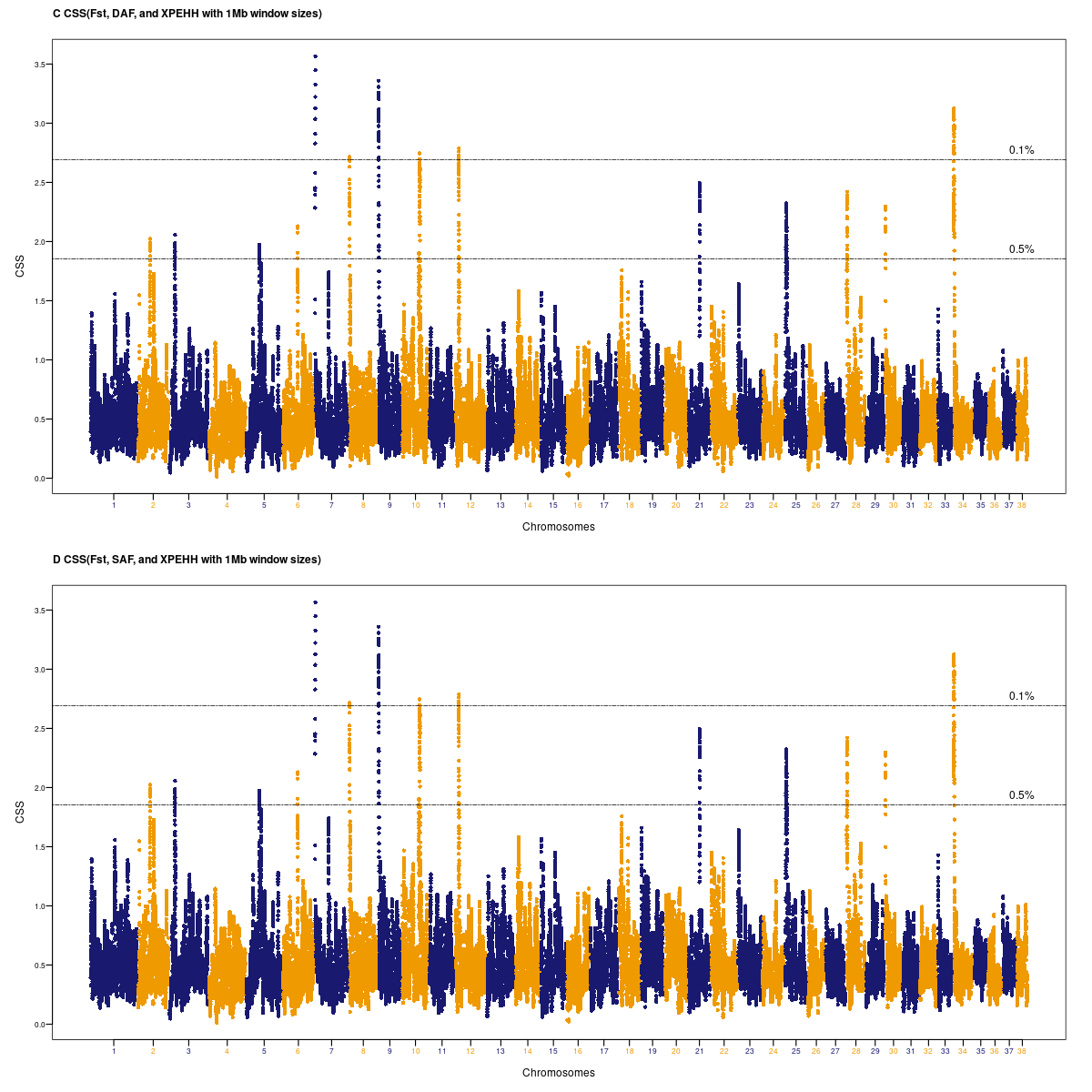


Supplementary Figure S1. Manhattan plots of various genome scans for Qinghai-Tibetan Plateau dogs A-D, the results from CSS (*Fst*, ∆DAF or ∆SAF and XP-EHH) were shown in A-D. The black lines show the threshold for genome-wide significance (top 0.1% of ranking and top 0.5% of ranking, respectively).


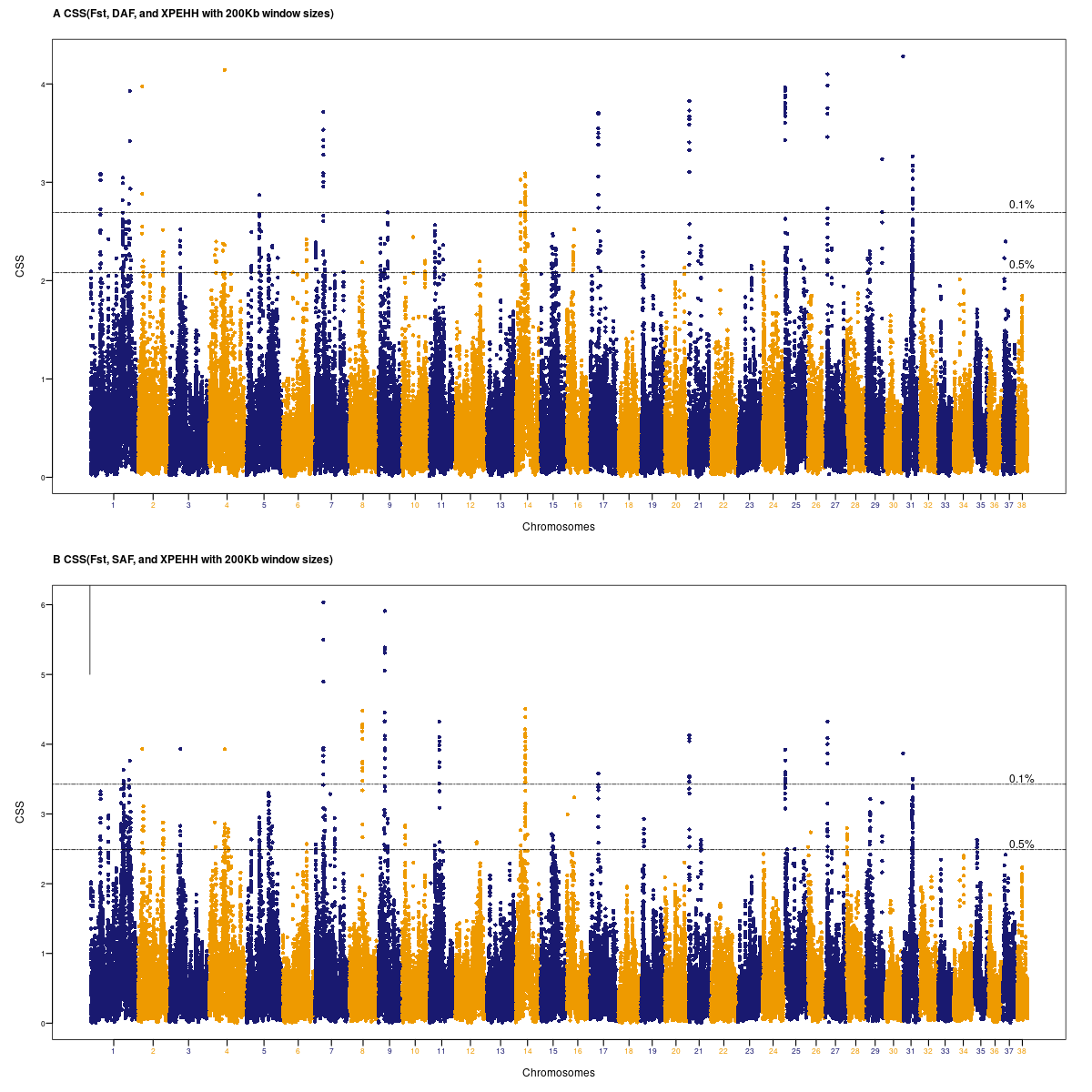


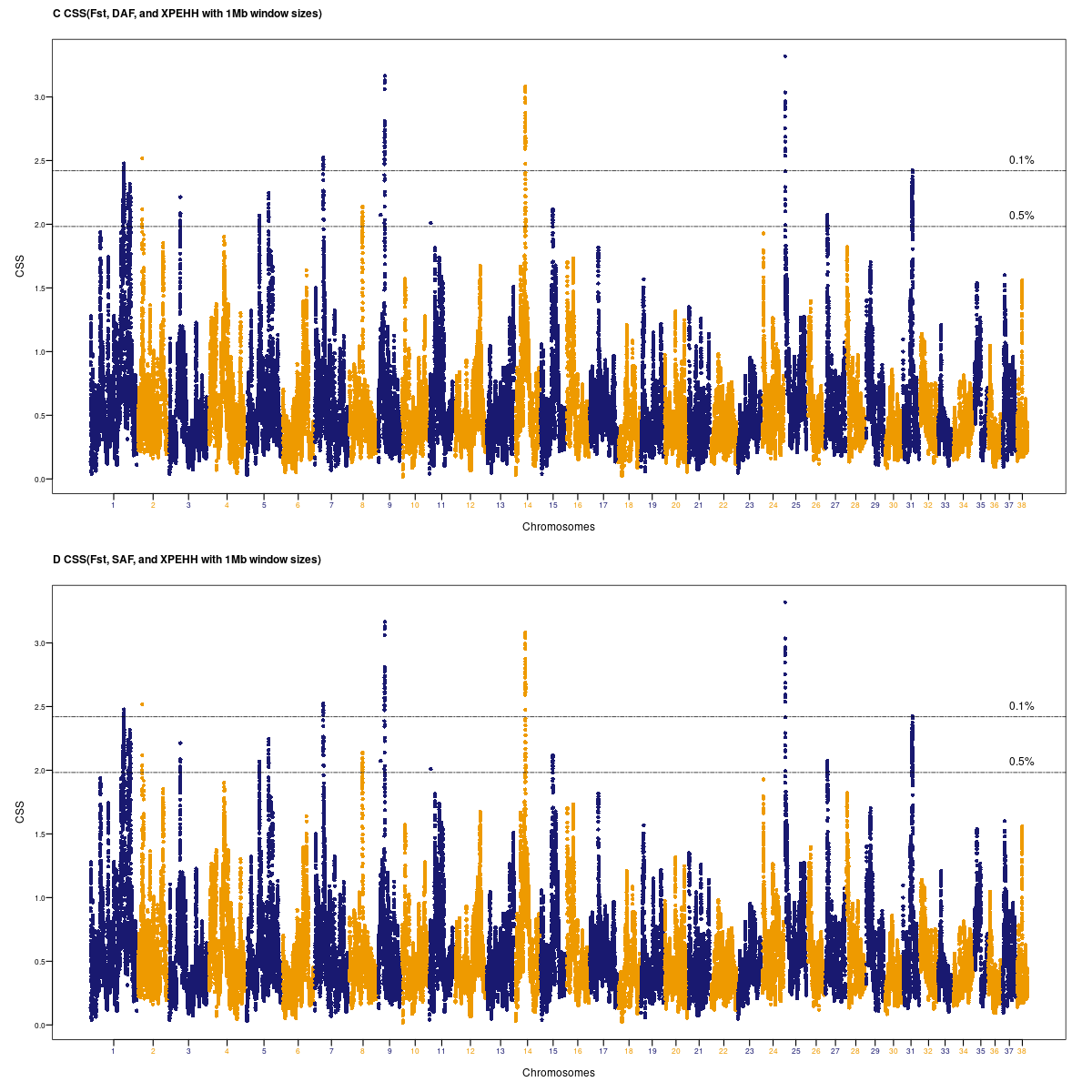


Supplementary Figure S2. Manhattan plots of various genome scans for Xi dogs A-D, the results from CSS (*Fst*, ∆DAF or ∆SAF and XP-EHH) were shown in A-D. The black lines show the threshold for genome-wide significance (top 0.1% of ranking and top 0.5% of ranking, respectively).


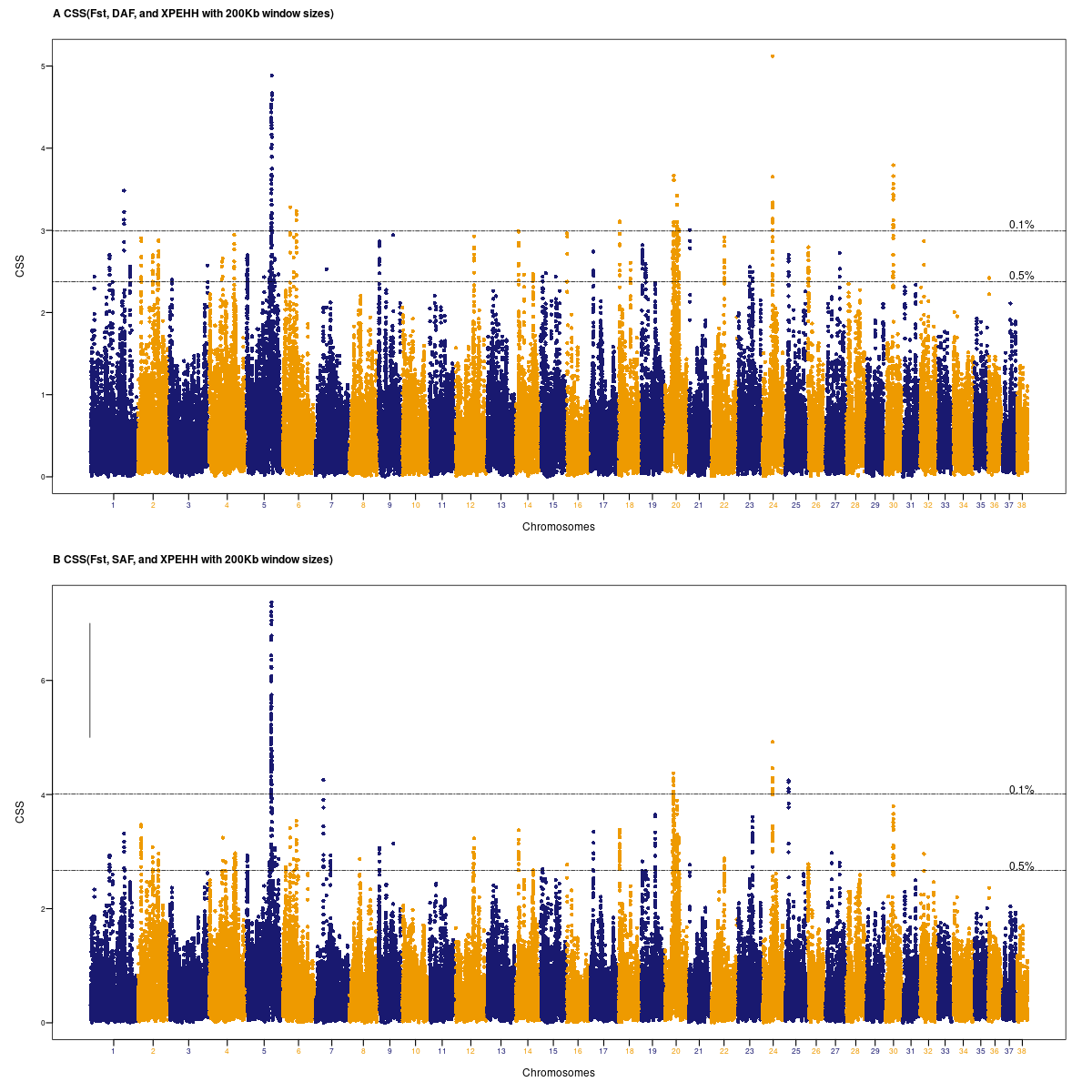


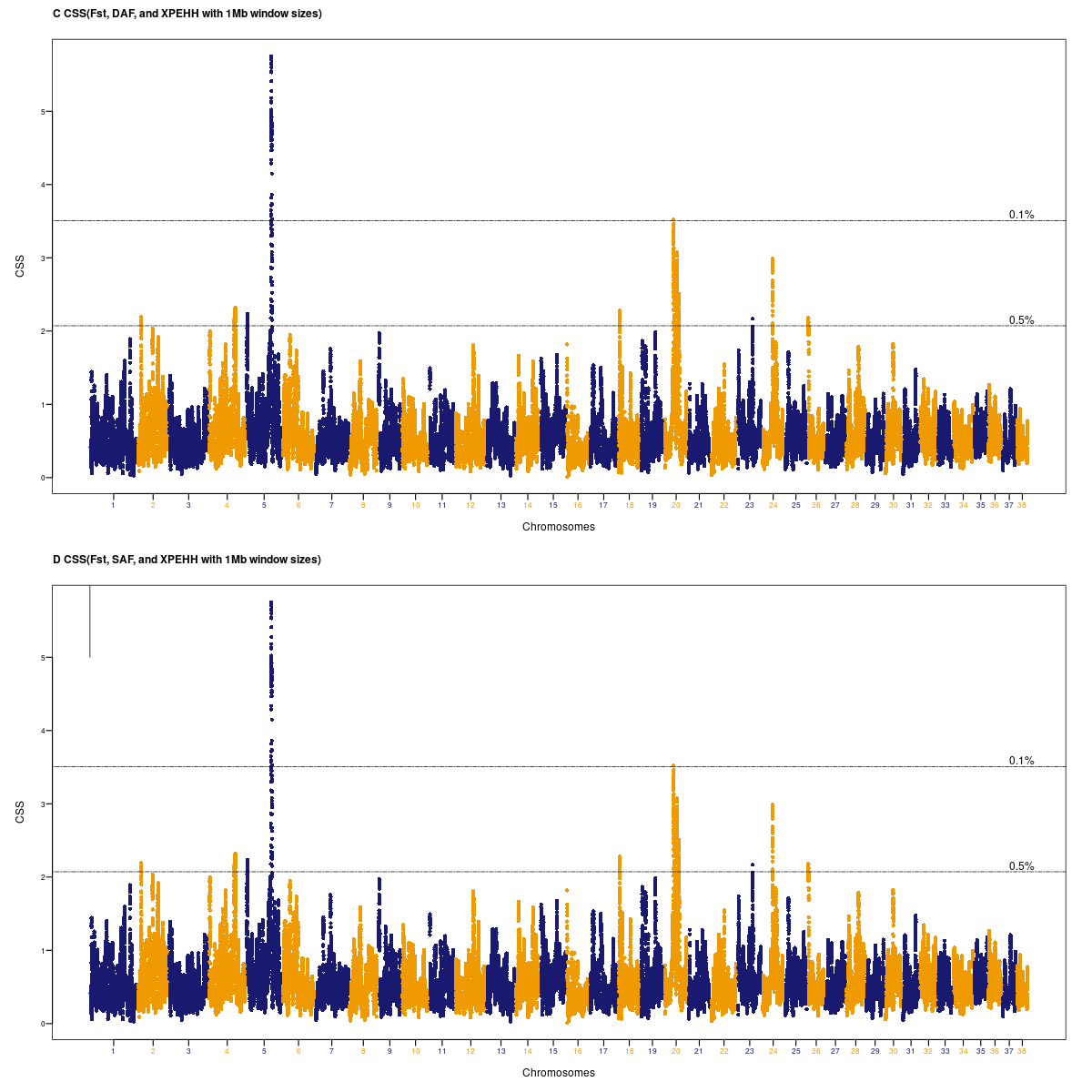


Supplementary Figure S3. Manhattan plots of various genome scans for Mountain hounds A-D, the results from CSS (*Fst*, ∆DAF or ∆SAF and XP-EHH) were shown in A-D. The black lines show the threshold for genome-wide significance (top 0.1% of ranking and top 0.5% of ranking, respectively).
