# Supplementary material for: Analysis of dog breed diversity using a composite selection index
Source: Sci Rep. 2023 Jan 30;13:1674. doi: 10.1038/s41598-023-28826-3 (PMC9886904; doi:10.1038/s41598-023-28826-3)
Supplement: Supplementary file 14 — Supplementary Information 14. [file 41598_2023_28826_MOESM14_ESM.docx]

Supplementary Table S13. Grouping of dogs for CSS analysis. The Tibetan Mastiff, Hequ Tibetan Mastiff and Linzhi dog were grouped as plateau dog breeds. Shandong Xi dog and Shaanxi Xi dog were merged and used for a target group with fast running speed (60 km/h). The Liangshan and Qingchuan hounds were classified on the basis of exceptional hunting ability.

| Criteria used to define the group target | No. of dogs | Breed (N) | Criteria used to define Reference group | No of reference dogs | Reference Breed (N) |
| --- | --- | --- | --- | --- | --- |
| High altitude (altitude > 3000 meters located in plateau) | 34 | Hequ Tibetan mastiff (7), Tibetan mastiff (15), Linzhi dog (12), | Low altitude (altitude < 1000 meters located in non-plateau) | 133 | Chuandong hound (12), Kazakhstan shepherd dog (4), Mongolia Xi dog (12), Chinese country dog (9), Guangxi hound (12), Liangshan hound (12), Pekingese (3), Qingchuan hound (12), Shandong Xi dog (12), SharPei (21), Shanxi Xi dog (12), Xiasi hound (12) |
| running speed (Xi dogs) | 24 | Shandong Xi dog (12), Shanxi Xi dog (12) | Non-Xi dogs | 124 | Chuandong hound (12), Kazakhstan shepherd dog (4), Chinese country dog (9), Guangxi hound (12), Tibetan mastiff (15), Liangshan hound (12), Pekingese (3), Qingchuan hound (12), SharPei (21), Linzhi dog (12), Xiasi hound (12) |
| Hunting ability (Mountain hounds) | 24 | Liangshan hound (12), Qingchuan hound (12) | Non-Mountain hounds | 70 | Kazakhstan shepherd dog (4), Mongolia Xi dog (12), Tibetan mastiff (15), Pekingese (3), Linzhi dog (12), Shandong Xi dog (12), Shanxi Xi dog (12) |
